# What Can Mobile Sensing and Assessment Strategies Capture About Human Subjectivity?

**DOI:** 10.3389/fdgth.2022.871133

**Published:** 2022-04-15

**Authors:** Bruno Biagianti

**Affiliations:** ^1^Department of Pathophysiology and Transplantation, University of Milan, Milan, Italy; ^2^Department of Neurosciences and Mental Health, Fondazione IRCCS Ca' Granda Ospedale Maggiore Policlinico, Milan, Italy

**Keywords:** mobile health (mHealth), EMA assessment, passive sensing, digital health, mobile interventions

## Introduction

The pervasiveness of mobile technology on the way we learn, create, work, share, communicate, move, love, and live today exceeds our individual ability to choose whether to be affected or not by it ^1^. The unique degree to which each of us is exposed has profound implications on the behavioral responses we are able to generate, with adaptation being increasingly considered the most welcome ^2^.

Medical knowledge and clinical care are being revolutionized by mobile technology, even more so after the onset of the COVID-19 pandemic, which has functioned as an accelerator of changes that have permanently reshaped the way providers and patients today think of, talk about, and manage physical and mental health (1).

The relationship between mental health and mobile technology is growing more complex and unpredictable every day, with providers and researchers animated both by the drive to keep up with the promising tools developed by technological innovation, and the need to better characterize and understand this relationship prior to taking actions that may prove all but therapeutic in the long term (2, 3). A controversial area of debate that has emerged from the relationship between mental health and technology is whether the unprecedented wealth of data captured by a mobile device can produce information that ultimately has clinical relevance for the provider and/or for the patient, and whether the acquisition, understanding, and attribution of meaning to said information comes with a toll (4, 5). Addressing the latter point likely offers tools to approach the former with greater awareness.

## Sensing and Measuring the Ecological Self

Reviewing the physical and psychological implications of being tethered on human perception, consciousness, and behavior exceedingly falls outside the scope of this letter (6). Suffice to say that psychological constructs such as boundaries, once considered indispensable for shaping subjective identity and demarcating it from external reality—or at least a shared definition of it—are being interrogated if not questioned by mobile technology (7). Mobile sensing—i.e., technology that passively collects data from sensors embedded in mobile devices—arguably represents the epitome of this transformation. Modern day smartphones come with a number of embedded sensors such as a high-resolution complementary metal-oxide semiconductor, image sensor,^1^,^2^ global positioning system (GPS) sensor, accelerometer, gyroscope, magnetometer, ambient light sensor, and microphone (8). These sensors can be used to measure several health parameters such as heart rate (HR), HR variability, respiratory rate, sleep quality, and health conditions such as ear and eye diseases, thus turning the smartphone into a continuous and long-term health monitoring system (9). Moreover, using a combination of data from motion sensors and cellular network providers, today it is rather trivial to capture movement patterns—including step count, time spent inside and outside, distance traveled. These variables all serve as proxies of physical daily activity levels, and are known to predict and impact mental wellbeing (10). Similarly, frequency and length of SMS and calls stored on the device that have been shown to function as proxies of social activity levels (11). Even more so than with Ecological Momentary Assessments (i.e., repeated sampling of subjects' current behaviors and experiences in real time, in subjects' natural environments)—which naturally pose issues of engagement, reliability, and durability—mobile sensing demonstrates that as contemporary subjects we are effortlessly traceable, measurable, and most importantly, knowable in ways to which we have been historically oblivious (12–14). Yet, enthused by the opportunities that mobile technology is presenting, we have perhaps paid less attention to the relevance of what exactly is being known of us (15, 16).

## Discussion

The matter of signifying incoming data transmitted in real time by mobile devices must not cease to interpellate researchers, clinicians and end-users likewise (17). If artificial intelligence and machine learning algorithms today allow quite strikingly to chart and predict the fluctuations of measured behaviors, should there exist an interdisciplinary dialogue on the epistemological responsibilities of this process? Certainly, the medical field has long resorted to the measurement of quantitative variables in order to provide patients with clinical information, yet the infrequent collection of such measurements, along with the interpretation and delivery of their findings, has been a prerogative of trained health professionals. The ease with which enormous amounts of data can be captured and transmitted by mobile devices today, in addition to the fact that mobile sensing and real-time assessments minimize recall bias, maximize ecological validity, and allow study of micro-processes that can influence real-world behavior, are truly dismantling the existing and collectively-shared definitions of health and illness (18), while shedding new, slightly darker, light of scientific positivism (19). If “the more we measure, the more we know” has induced unimaginable progress and innovation in the medical field thus far, now that we realize how much technology can measure, can we feel equally confident about “the more we measure, the more we understand,” especially in the delicate matter of subjectivity (20)?

The definition of subjectivity and its implications on the self-perceived relationship between health and illness has entertained different psychological theorists. Just to list a few, psychoanalysis, Gestalt and post-structuralist approaches have all attempted a specific proposition that reflects distinct cultural-historical traditions (21). In its simplest and most generous connotation, subjectivity is the first-person perspective, no more and no less. The closet concept that cognitive sciences have coined is self-reference, which describes the way a particular individual perceives itself in relation to its immediate environment and its total life situation (22). Certainly, a current viewpoint exists and takes form when a relationship is formed between a subject and its object, between observer and observed. Nonetheless, while acknowledging that a viewpoint could never be described as belonging to a person in any enduring sense, systematic investigations of personality show recurrent traits, observation styles that tell more about the ways in which the subject typically processes the object, whether that is a circumstance, an event, or some other kind of enquiry (23–25). It emerges that subjectivity includes emotionally loaded conceptions and symbolical processes that are generated throughout human experience, and determine, to a large degree, choices, actions and interactions, which all directly impact the degree of functioning and malfunctioning of any individual in its ecological system (26). As a result, the heuristic value of subjectivity encompasses processes that can neither be exhausted by language, nor by discourse, nor, at least not entirely, by awareness (27).

In this context, the matter of new orders of data about the psychological self mobilizes in both providers and patients hierarchically ordered conceptual categories and metacategories, which all express the subjective signification process (28). These categories should not be neglected, and form instead the basis for intersectional conversations between patients, clinicians, scientists and technological innovators on the personal and clinical values that such data should have (29). If historically objective science has been conducted prioritizing the third-person perspective over the first-person accounts, we must recognize that the third-person perspective no longer belongs to a person, but it is now being produced by technological advances^3^. The so-called data driven discoveries, at least in the field of mental health, are often eluding the critical processes of conceptualization, signification, and interpretation—processes that allow humans to grow and know themselves. More importantly, the unbridled proliferation of subject-specific behavioral mobile data is creating the illusion that the foundation for a science of subjectivity is being laid, whereas what is arguably occurring is that subjectivity, with its eschatological and existential functions (30)—functions that cannot and should not be measured, “scientified,” technologized—is being declassified.

After a decade of mobile mental health research, we hope that these considerations can enrich the current dialogue between key stakeholders (clinicians, researchers, patients, policy makers, funding agencies, and development firms) about the directions that the field of mobile sensing and assessments could explore moving forward. What kind of a research roadmap can be then proposed for the field? Possibly, one where critical aspects of human knowledge and experience that would largely lose their complexity and value if measured—aspects that cannot be absorbed into the mobile technology revolution—will continue to be studied, divulged, epistemologically dignified, and placed in a position of dialectical interplay with technology-based knowledge.

## Author Contributions

The author confirms being the sole contributor of this work and has approved it for publication.

## Funding

This work was funded through the Fondazione Regionale Ricerca Biomedica under the Marie Skłodowska-Curie Actions Seal of Excellence fellowship (ID 2681424) awarded to BB.

## Conflict of Interest

BB serves as a consultant for Posit Science, a company that produces mobile assessment software.

## Publisher's Note

All claims expressed in this article are solely those of the authors and do not necessarily represent those of their affiliated organizations, or those of the publisher, the editors and the reviewers. Any product that may be evaluated in this article, or claim that may be made by its manufacturer, is not guaranteed or endorsed by the publisher.

## Footnotes

^1^ https://www.pewresearch.org/internet/2018/04/17/the-future-of-well-being-in-a-tech-saturated-world/

^2^ https://sloanreview.mit.edu/article/pushing-back-on-pervasive-technology/

^3^ https://techcrunch.com/2016/11/18/big-data-cant-bring-objectivity-to-a-subjective-world/
